# Systematic Mining of Bioactive Compounds for Wound Healing From Cayratia Japonica Exosome-Like Nanovesicles: A Workflow Combining LC-MS and DeepSeek Models

**DOI:** 10.2196/80539

**Published:** 2026-01-08

**Authors:** Qiang Fu, Wei Ji, Yu-Ping Fan, Jian Yao, Ming-Xia Song, Qiao-Jing Yan

**Affiliations:** 1 School of Basic Medical Sciences, Jinggangshan University, Ji'an, China; 2 Jiangxi Province Key Laboratory of Organ Development and Epigenetics, Clinical Medical Research Center, Affiliated Hospital of Jinggangshan University, College of Jinggangshan University, 28 Xueyuan Road, Qingyuan District, Ji'an, 343009, China, 86 07968100735; 3 University of Montpellier, Montpellier, France; 4 Department of Epidemiology & Biostatistics, School of Public Health, Southeast University, Nanjing, China; 5 Division of Molecular Signaling, Department of the Advanced Biomedical Research, Interdisciplinary Graduate School of Medicine, University of Yamanashi, Chuo, Japan; 6 College of Traditional Chinese Medicine and Pharmacy, Jinggangshan University, Ji'an, China

**Keywords:** DeepSeek, liquid chromatography-mass spectrometry, LC-MS, *Cayratia japonica* exosome-like nanovesicles, CJ-ELNs, artificial intelligence, AI-powered multimodal framework, wound healing and tissue regeneration

## Abstract

**Background:**

Plant-derived exosome-like nanovesicles (P-ELNs) effectively deliver bioactive compounds due to their high biocompatibility and low immunogenicity. While liquid chromatography-mass spectrometry (LC-MS) profiles compounds in complex samples, its analysis of large datasets remains limited by traditional methods. Recent advances in large language models (LLMs) and domain-specific systems have enhanced Chinese biomedical data processing and cross-modal pharmaceutical research.

**Objective:**

This study aimed to create a multimodal framework of LC-MS combined with DeepSeek models for data mining of compounds with wound-healing properties from exosome-like nanovesicles derived from *Cayratia japonica* (CJ-ELNs).

**Methods:**

LC-MS identified compounds enriched in CJ (n=3) and CJ-ELNs (n=3), and then compounds specifically enriched in CJ-ELNs were filtered via a four-step filtering workflow. The CJ-ELNs-specific compounds were processed by DeepSeek models for screening naturally active compounds with targeted functions of antioxidation, anti-inflammation, anticellular damage, antiapoptosis, wound healing and tissue regeneration, and cell proliferation.

**Results:**

A multimodal framework of LC-MS combined with the DeepSeek-DF model was created. With the assistance of artificial intelligence (AI), a total of 46 naturally active compounds derived from CJ-ELNs with targeted functions were identified.

**Conclusions:**

A self-designed multimodal framework of LC-MS, combined with DeepSeek models, rapidly and accurately identifies naturally active compounds from CJ-ELNs. This AI-powered system innovatively integrates the traditional analytical technique with modern LLMs, thus greatly favoring data mining of active ingredients in traditional Chinese medicine herbs.

## Introduction

Plant-derived exosome-like nanovesicles (P-ELNs) contain abundant bioactive molecules, serving as novel carriers of natural products to mediate intercellular communication and mediate physiological processes [1,2]. P-ELNs are superior to conventional mammalian-derived exosomes, possessing unique advantages such as high biocompatibility, high skin permeability, low cytotoxicity and low immunogenicity [3,4]. Multiple in vitro and in vivo studies indicate that these P-ELNs possess intrinsic therapeutic activity, offering promise for disease treatment and enhancing human health [5,6]. *Cayratia japonica*, a traditional Chinese medicinal herb, is widely used for the treatment of traumatic injuries such as contusions and lacerations [7]. Recent clinical studies have confirmed that topical application of CJ ointment effectively alleviates local inflammation and promotes the repair and regeneration of damaged tissue, demonstrating favorable therapeutic outcomes in the management of postoperative infectious wounds around the anus [8]. However, research and application of exosome-like nanovesicles (ELNs) derived from CJ remain incomplete. Our research team successfully extracted and characterized a novel type of P-ELNs from the traditional Chinese medicinal herb *Cayratia japonica*, namely *Cayratia japonica* exosome-like nanovesicles (CJ-ELNs). They possess efficient delivery of bioactive compounds to wound sites, thus favoring tissue regeneration from infectious wound-related disorders. Bioactive constituents encapsulated within CJ-ELNs are dominant in wound healing. Consequently, the identification and characterization of bioactive compounds responsible for wound healing are of paramount significance.

Great strides have been made in the screening of active ingredients from natural products via omics techniques [9]. Liquid chromatography–mass spectrometry (LC-MS) has emerged as a powerful tool for profiling trace-level compounds in complex samples, although its performance in processing massive data is limited by traditional manual or rule-based analytical approaches [10,11]. In recent years, large-scale pretrained language models (LLMs), such as ChatGPT, GPT-4, and domain-specific systems like DeepSeek, have significantly transformed the landscape of biomedical data analysis and knowledge discovery [11,12]. These models exhibit powerful capabilities in natural language understanding, semantic reasoning, and prompt-based knowledge retrieval [13-15]. They are promising tools to assist omics analysis. In particular, DeepSeek models have been widely adopted for optimizing Chinese-language biomedical contexts, and supporting cross-modal tasks in pharmaceutical research, such as entity recognition, document summarization, and semantic ranking [16,17].

In this study, we innovatively created a multimodal framework of LC-MS combined with DeepSeek models for data mining of compounds with wound-healing properties from CJ-ELNs. This work illustrates the potential of artificial intelligence (AI) as a computational engine in natural compound discovery and offers a scalable solution for mining multimodal biochemical data.

## Methods

### Preprocessing of LC-MS Data

**Figure 1.**
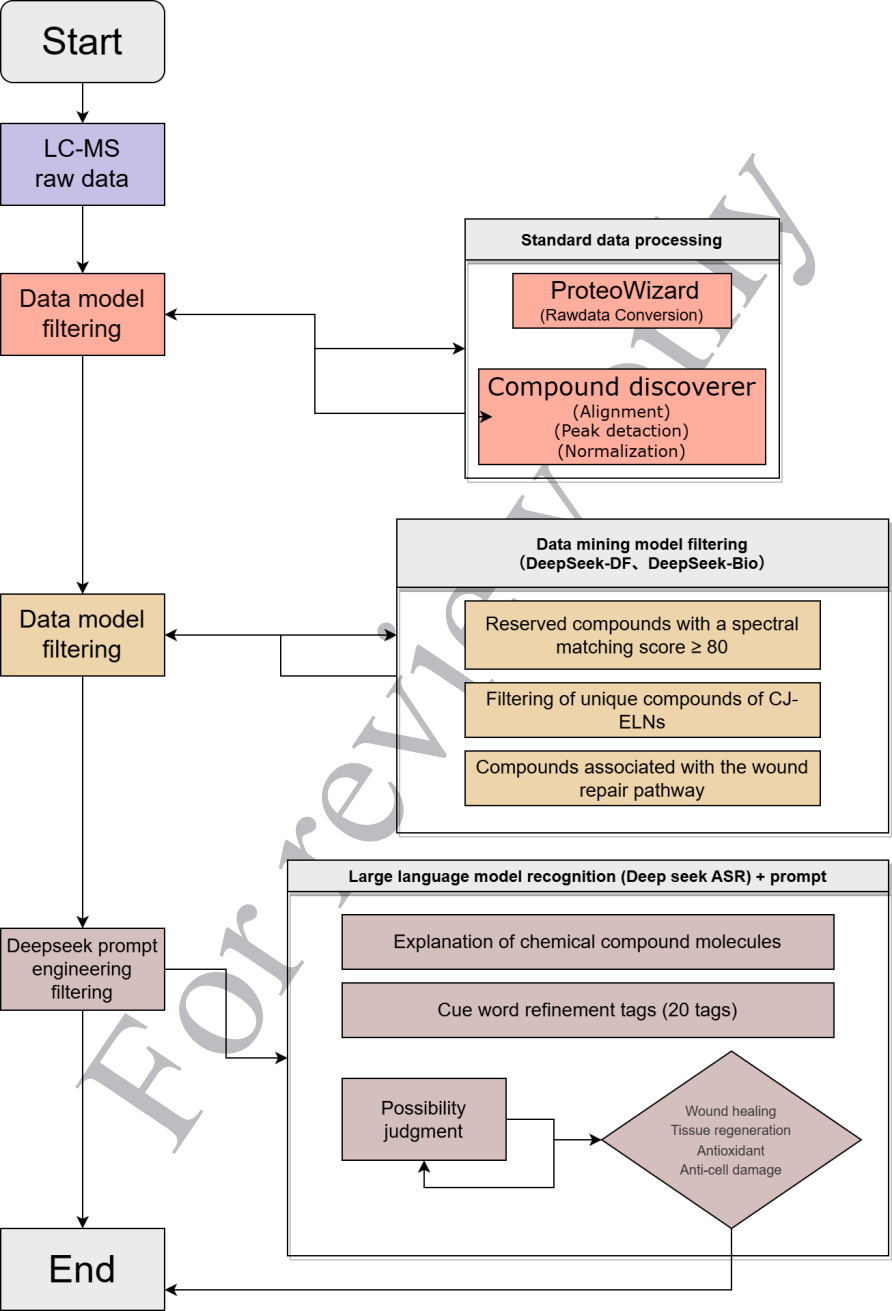
A four-step filtering workflow. CJ-ELNs: *Cayratia japonica* exosome-like nanovesicle; LC-MS: liquid chromatography-mass spectrometry.

Untargeted metabolomic profiling of CJ and CJ-ELNs was performed by LC-MS. A total of 6 samples (including 3 CJ samples and 3 CJ-ELNs samples) were analyzed using a ultra-high-performance liquid chromatography (UHPLC) system coupled to a Q Exactive HF-X mass spectrometer (Thermo Scientific). Chromatographic separation was performed on an HSS T3 column (maintained at 40°C) with a 12-minute linear gradient from 2% to 98% mobile phase B at a flow rate of 0.3 mL/min. Mass spectrometry (MS) data were acquired in both positive and negative electrospray ionization (ESI) mode (± ESI) using a data-dependent acquisition strategy (top 10 most intense ions). Raw data were first converted to the mzML format using ProteoWizard, followed by processing, using Compound Discoverer 3.3 (Thermo Fisher Scientific) for peak alignment (with maximum retention time shift of 0.5 min and mass tolerance of 10 ppm) and normalization (using the median of maximum peak areas). Compound identification was achieved by matching MS/MS spectra against the following databases: mzCloud, LipidMaps, KEGG, HMDB, and MassBank. The matching criteria were set to a mass tolerance of 10 ppm and a minimum match factor threshold of 10. A four-step filtering workflow was designed to quantitatively identify target compounds as follows (Figure 1).

1. Filtering of match confidence: compounds with spectral match scores ≥80 were retained [18];
2. Filtering of unique compounds of CJ-ELNs: compounds identified in CJ and CJ-ELNs were compared with isolated compounds unique to CJ-ELNs;
3. Filtering of biological relevance: candidate compounds were screened for associations with wound healing-related signaling pathways using the DeepSeek-Bio model;
4. Semantic recognition and prompt engineering: final candidate molecules were refined through semantic analysis and prompt-based selection.

Common and unique compounds derived from CJ and CJ-ELNs were visualized in a Venn diagram, and a word cloud analysis was conducted via Python. Functions and tools, and databases of key terms used in this study are listed in Table 1.

**Table 1.** Key terms, functions, tools and databases used in this study.

Key terms	Functions	Tools/databases
mzML	Standardized data storage	ProteoWizard
DeepSeek-Bio	Biological pathway association analysis	Deepseek 671B Model Network Edition KEGG database
Morgan	Digital characterization of molecular structures	Chemoinformatics software packages
PubMedBERT	Literature feature extraction	PubMed.pro
Grad-CAM	Visualization of model decisions	Deep learning frameworks (eg, PyTorch)
ASR	automatic semantic recognition	The Great Prophecy Model of Human-Computer Interaction

### Construction of a Multimodal Framework of LC-MS Combined With DeepSeek Models

**Figure 2.**
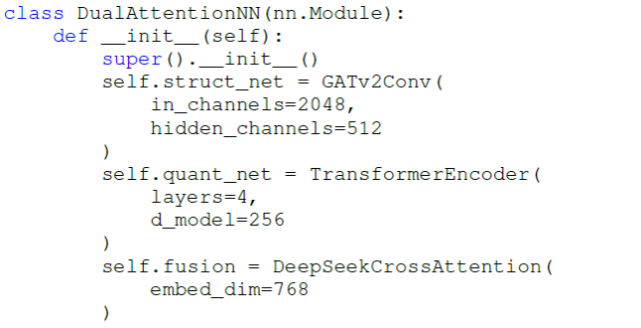
The core architecture of the input layer.

A multimodal framework of LC-MS combined with the DeepSeek-DF model was created, consisting of two major components of the input and output layers. The input layer integrated structural features of compounds (Morgan fingerprints), quantitative features (*z* score normalization), and literature-derived features (PubMedBERT embeddings). The core architecture was listed in Figure 2. Additionally, the output layer used multitask learning to simultaneously predict wound-healing activity via Sigmoid output and mechanism category via Softmax output.

### Interpretability-Based Filtering

The Automated Semantic Recognition (ASR) module and prompt engineering techniques of DeepSeek-R1 32B, as well as web searching were used to interpret the potential biological functions of the screened candidate compound with an annotation of functional labels. A plausibility assessment was then performed based on predefined criteria, including antioxidation, anti-inflammation, anticellular damage, antiapoptosis, wound healing and tissue regeneration, and cell proliferation. Each compound was evaluated and categorized using the following scoring scheme: √ (confirmed), × (not supported), and ? (uncertain). Taking the metabolite (-)-Epicatechin 3-O-gallate as an example, its function, category and possibility in the involvement of wound healing, tissue regeneration, antioxidant, and anticellular damage were predicted via the multimodal framework (Table 2). Following this preliminary filtering, manual curation was conducted to eliminate compounds of nonplant origin and those with low abundances. Ultimately, a refined set of characteristic natural products from CJ-ELNs with potential wound-healing properties was selected.

**Table 2.** Functions, categories and possibility in the involvement of biological processes of representative metabolites.

Compound	Functions	Categories	Possibility
(-)-Epicatechin 3-O-gallate	Antioxidant, anti-inflammatory, anti-cancer, cardiovascular protection, glucose and lipid metabolism regulation.	Organic compound, antioxidant factor, anti-inflammatory factor, energy metabolism, phenolic factor	Wound healing: ×, tissue regeneration: ×, antioxidant: √, anti-cellular damage: ?
Rutin	Antioxidant and anti-inflammatory, maintaining vascular resilience, reducing vascular permeability and fragility, exhibiting certain antiviral and anticancer effects.	Flavonoids, antioxidant, anti-inflammatory	Wound healing: ×, tissue regeneration: ×, antioxidant: √, anti-cellular damage: ?
Caffeine	Central nervous system stimulants, enhance mental alertness, alleviate fatigue.	Organic compounds, alkaloids, energy metabolism	Wound healing: ×, tissue regeneration: ×, antioxidant: ×, anti-cellular damage: ×

## Results

### Acidic Compounds Are Enriched in CJ-ELNs

**Figure 3.**
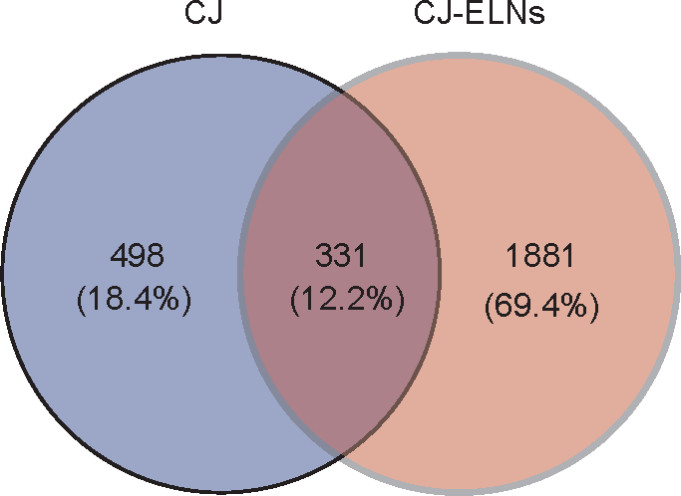
Enrichment of acidic compounds in CJ-ELNs. (A) A Venn diagram visualizing an intersection of compounds identified from both CJ and CJ-ELNs and unique compounds in CJ-ELNs. CJ: *Cayratia japonica*; CJ-ELNs: *Cayratia japonica* exosome-like nanovesicle.

After conversion and normalization of the raw LC-MS data, a total of 829 and 2212 compounds were identified from CJ and CJ-ELNs. A Venn diagram visualized 1881 specific compounds in CJ-ELNs (Figure 3). “Acid,” as the most frequent term across all entries of metabolite names, was detected by a word cloud analysis (Multimedia Appendix 1). It suggested that acidic compounds were highly enriched in CJ-ELNs.

### Rapid and Accurate Data Mining of Compounds in CJ-ELNs With Functional Properties

**Figure 4.**
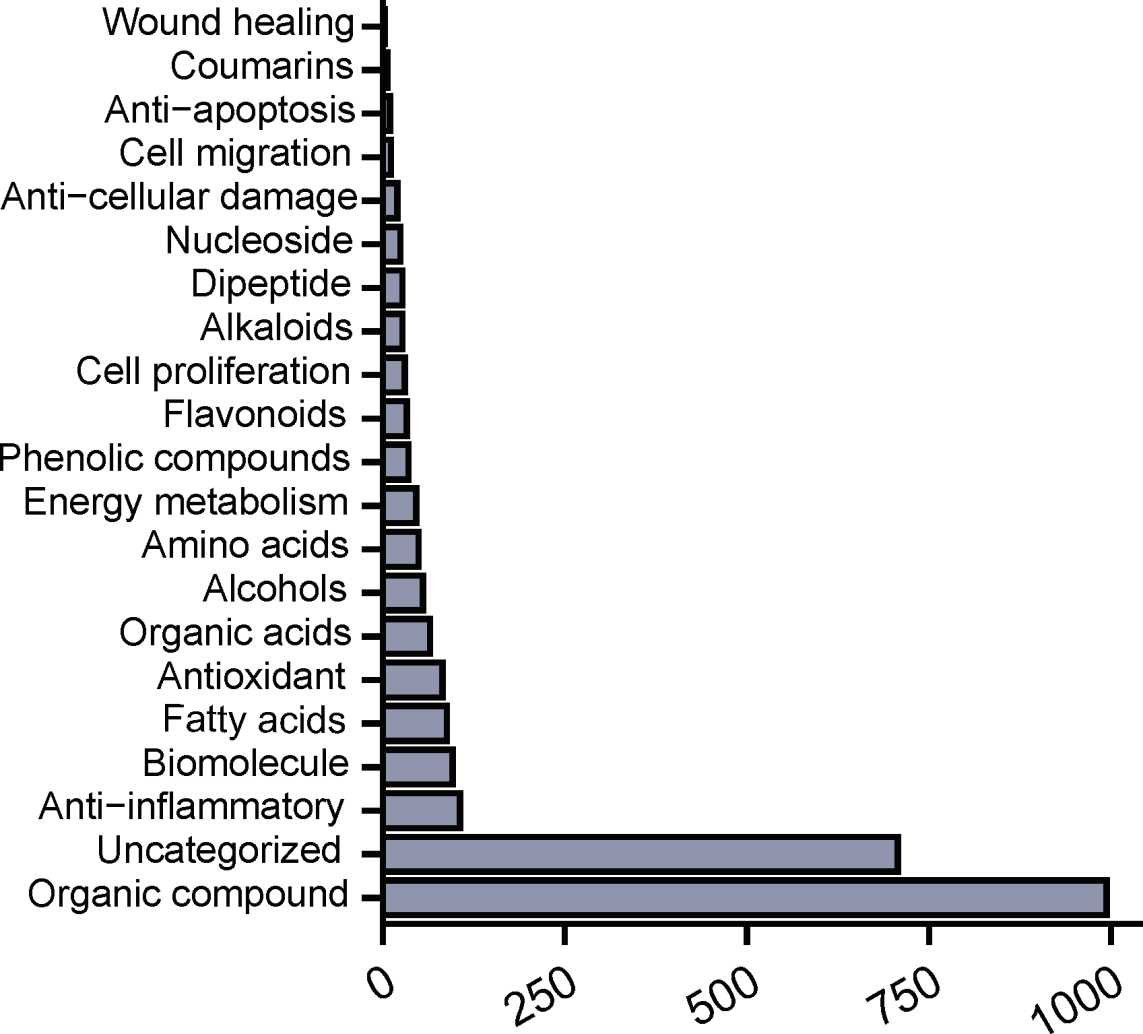
Rapid and accurate data mining of compounds in CJ-ELNs with functional properties. Top 20 classifications of compounds enriched in CJ-ELNs. CJ-ELN: *Cayratia japonica* exosome-like nanovesicle*.*

A total of 1881 candidate compounds enriched in CJ-ELNs were functionally annotated and classified using the self-designed multimodal framework of LC-MS combined with DeepSeek models. They were categorized into 20 distinct classes, including organic compounds, alkaloids, amino acids, biomolecules, organic acids, antioxidants, anti-inflammatory agents, energy metabolism-related molecules, phenolics, cytoprotective agents, alcohols, and others. Organic compounds were the leading category of compounds enriched in CJ-ELNs (Figure 4, Multimedia Appendix 2). Functionally, 43.33% (n=39) of compounds enriched in CJ-ELNs possessed the antioxidant property. With the assistance of DeepSeek, we specifically screened compounds enriched in CJ-ELNs with targeted functions of antioxidation, anti-inflammation, anticellular damage, antiapoptosis, wound healing and tissue regeneration, and cell proliferation.

### Bioactive Compounds of CJ-ELNs Responsible for Wound Healing and Tissue Regeneration

**Figure 5.**
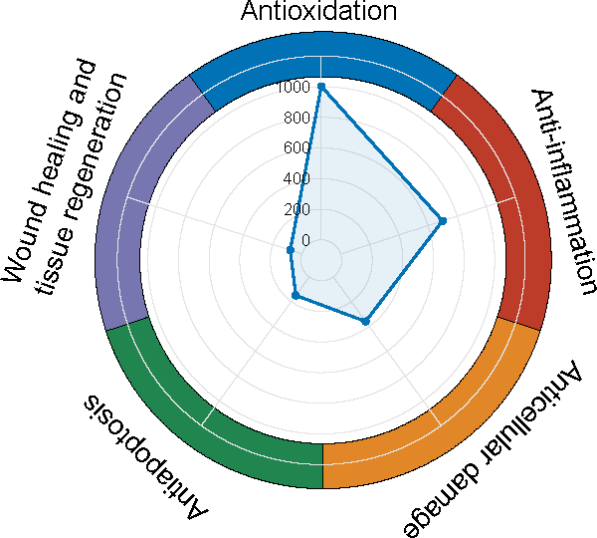
Radar plots visualizing bioactive compounds of *Cayratia japonica* exosome-like nanovesicles with targeted functions.

**Figure 6.**
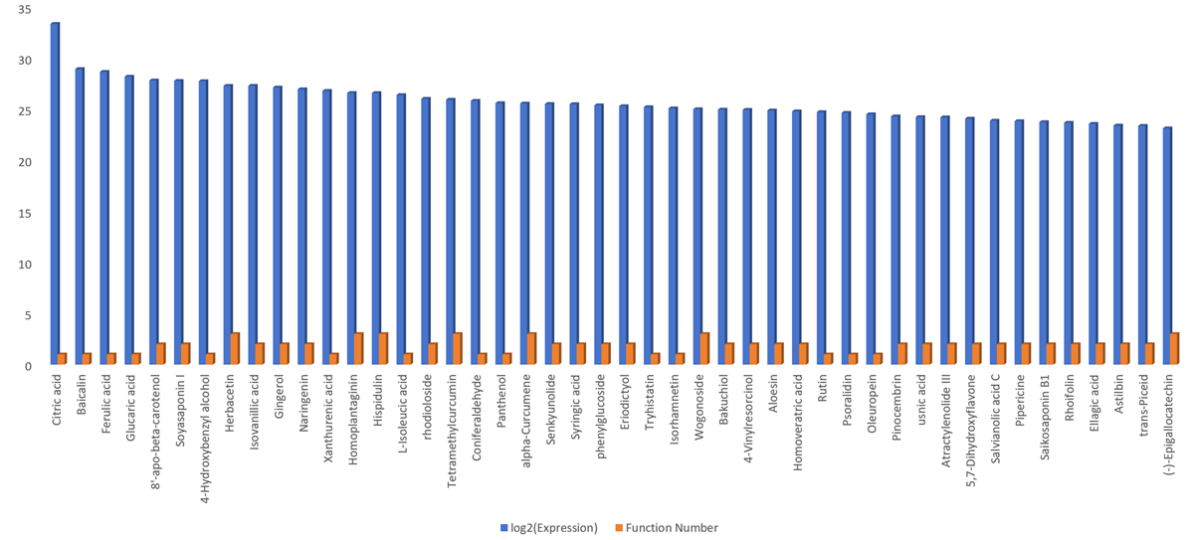
Expression levels (log_2_-transformed) of naturally active compounds derived from *Cayratia japonica* exosome-like nanovesicle identified by an integration of liquid chromatography-mass spectrometry and DeepSeek models.

We estimated the overall expression levels of compounds across the six target functions derived from the DeepSeek model within this multimodal framework, visualizing the results in radar chart format after log2-transformation. (Figure 5). Notably, compounds with the antioxidant function possessed the highest expression levels, proving the antioxidant mechanism of CJ-ELNs in wound repair. Finally, a secondary filtering of compounds with targeted functions was conducted. We manually excluded nonplant–derived compounds, including those of animal origin, synthetic chemicals, and other nonbotanical sources. In addition, compounds with low expression levels in CJ-ELNs were also removed. As a result, a total of 46 naturally active compounds derived from CJ-ELNs with targeted functions were identified (Figure 6 and Multimedia Appendix 3). Citric acid was the most abundant compound with the targeted functions, which was consistent with the finding from the word cloud analysis.

## Discussion

### Principal Findings

This study innovatively integrated DeepSeek models with LC-MS to successfully predict the major natural products of CJ-ELNs responsible for wound healing. DeepSeek’s ASR semantic recognition and prompt engineering worked together to generate initial classification labels. Moreover, an automatic assessment effectively, rapidly, and accurately achieved the goal of data mining of specific compounds for targeted functions.

AI techniques, particularly LLMs, have become an unstoppable force for reshaping medical research [19,20]. Traditionally, LC-MS is a powerful analytical technique to identify and quantify active ingredients in traditional Chinese medicine (TCM) herbs. However, a rapid and accurate recognition of compounds with targeted functions, and a quantitative analysis of trace concentrations in complicated samples can be challenging [21]. We expected that an integration of LC-MS and LLMs would benefit TCM research, including the acceleration of active ingredient screen, precise targeting of interested compounds for certain diseases, and anchoring the promising candidates for developing new drugs. DeepSeek is an intelligent system based on a large-scale pre-trained language model, exhibiting strong capabilities in text understanding, knowledge reasoning, and cross-modal collaborative analysis, particularly excelling in processing information within Chinese-language contexts [22,23]. It enables rapid processing and analyzing massive volumes of both unstructured and structured data, thus digging biological insights out of complex omics datasets [24,25].

In the present study, we first created a four-step filtering workflow and quantitatively identified target compounds from CJ-ELNs by LC-MS. The cloud word analysis emphasized the term of acid among screened compounds enriched in CJ-ELNs. Acidic compounds derived from traditional Chinese herbals are established for the role of clearing heat and detoxifying [26]. Numerous studies have reported that acidic compounds in plants exert antioxidant, antibacterial, and anti-inflammatory effects through mechanisms such as scavenging free radicals, alleviating oxidative stress, modulating inflammatory factors, stimulating fibroblast proliferation, promoting collagen deposition, enhancing epithelialization, and inducing angiogenesis [27,28]. To achieve a precise data mining of compounds with relevant functions, DeepSeek models lent a hand that specifically screened compounds in CJ-ELNs with targeted functions of antioxidation, anti-inflammation, anticellular damage, antiapoptosis, wound healing and tissue regeneration, and cell proliferation. Finally, naturally active compounds in CJ-ELNs were resurfaced for their promising potentials in wound repair. For example, studies have shown that baicalin accelerates the wound healing process by downregulating the expression of pro-inflammatory cytokines (IL-6 and IL-1β) while upregulating the anti-inflammatory factor IL-10, and by promoting the secretion of various growth factors (VEGF, FGF-2, PDGF-β, and CTGF) [29]. The combination of LC-MS with DeepSeek paves the way to further analyses of therapeutic targets from traditional Chinese herbs for wound healing and tissue regeneration [30,31].

Limitations in this study should be noted. Firstly, bioactive compounds derived from CJ-ELNs were mined via LC-MS and a single LLM, namely, DeepSeek-R1. Other cutting-edge LLMs such as Claude, GPT-4 and Liama [32] can be further analyzed for the assistance of LC-MS in identifying interested compounds. Secondly, the 46 naturally active compounds derived from CJ-ELNs with targeted functions should be validated in in vivo and in vitro experiments. Lastly, the workflow we have established requires further validation on independent datasets. We shall address the aforementioned issues in subsequent work, including evaluating the efficacy of compounds through cell migration and transdermal tissue compatibility assays, verifying their efficacy via macroscopic imaging and H&E staining following animal wound modelling interventions, and validating potential pathways involved through Western blot and immunohistochemical analysis.

### Conclusion

We innovatively designed a multimodal framework of LC-MS combined with DeepSeek models that rapidly and accurately identify naturally active compounds from CJ-ELNs. This AI-powered system innovatively integrates the traditional analytical technique with modern large language models, showing a huge potential in modern medicine and TCM research.

## Supplementary material

10.2196/80539 Multimedia Appendix 1 A word cloud of common compounds identified by liquid chromatography-mass spectrometry.

10.2196/80539 Multimedia Appendix 2 Distribution of the classifications of compounds enriched in CJ-ELNs, distribution of functional compounds enriched in CJ-ELNs with targeted functions of wound healing and tissue regeneration, and distribution of compounds enriched in CJ-ELNs with all functional categories.

10.2196/80539 Multimedia Appendix 3 Function of 46 compounds.

## Abbreviations

AI: artificial intelligence
ASR: Automated Semantic Recognition
CJ-ELN: *Cayratia japonica* exosome-like nanovesicle
ELN: exosome-like nanovesicles
ESI: electrospray ionization
LC-MS: liquid chromatography-mass spectrometry
LLM: large language model
MS: mass spectrometry
P-ELN: Plant-derived exosome-like nanovesicle
TCM: traditional Chinese medicine
UHPLC: ultra-high-performance liquid chromatography
