# Supplementary figures and images for: Systematic Mining of Bioactive Compounds for Wound Healing From Cayratia Japonica Exosome-Like Nanovesicles: A Workflow Combining LC-MS and DeepSeek Models
Source: JMIR Bioinform Biotechnol. 2026 Jan 8;7:e80539. doi: 10.2196/80539 (PMC12784863; doi:10.2196/80539)

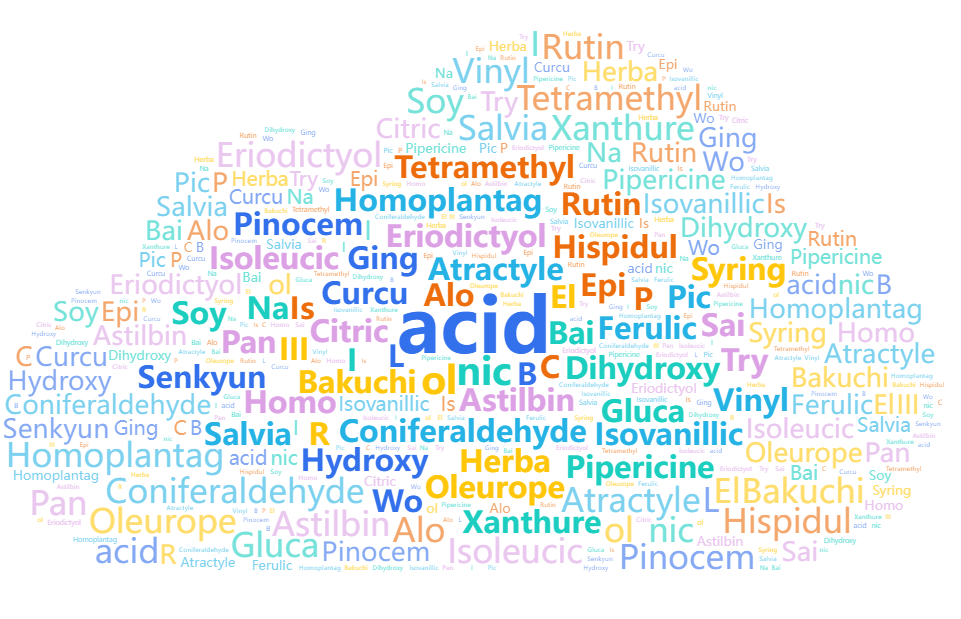

Supplement: Multimedia Appendix 1 [file bioinform-v7-e80539-s001.png]

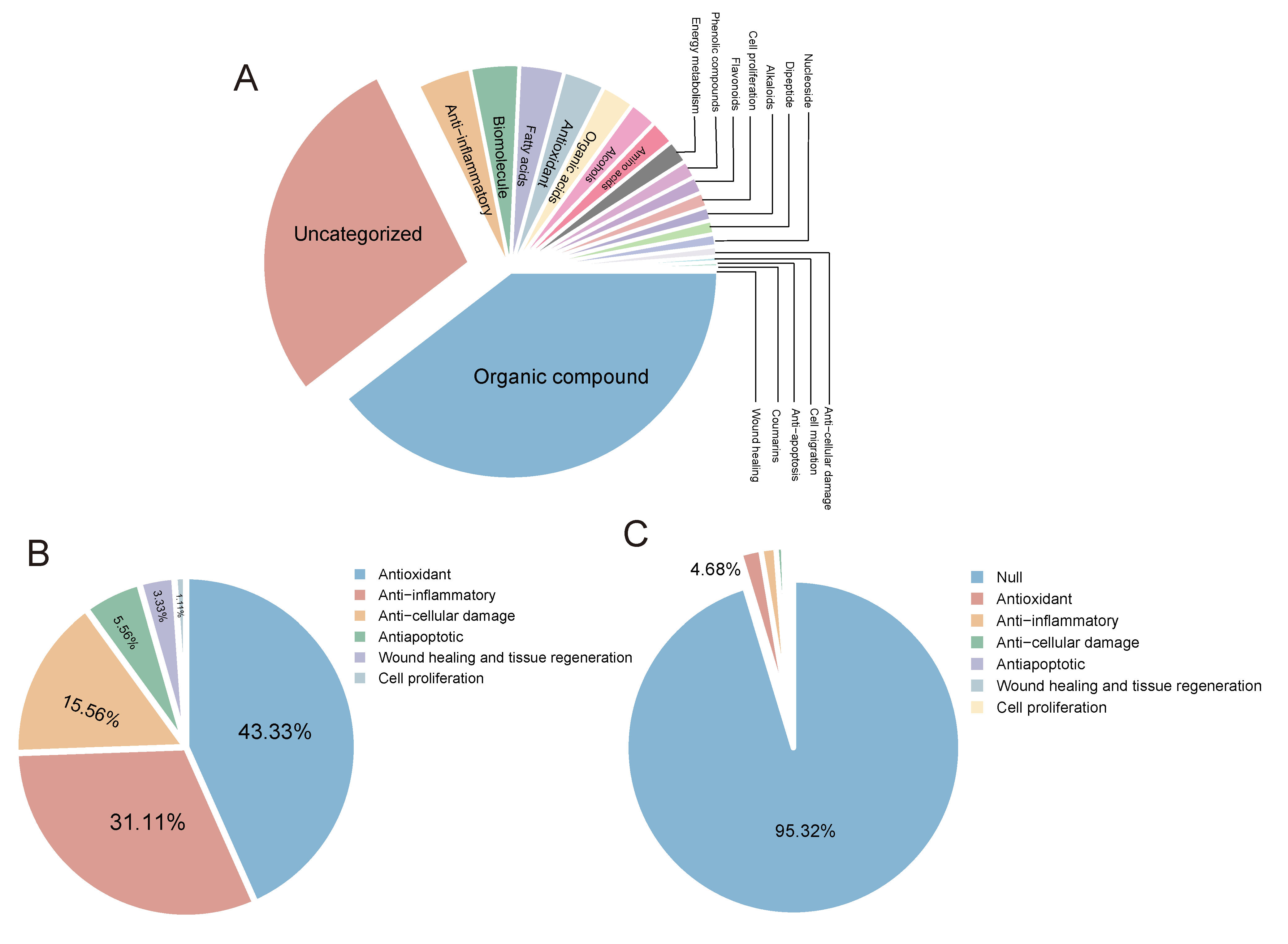

Supplement: Multimedia Appendix 2 [file bioinform-v7-e80539-s002.tif]
